# Experimental Duodeno-Cholecystostomy

**Published:** 1885-09

**Authors:** J. McF. Gaston

**Affiliations:** Atlanta, Georgia


### EXPERIMENTAL DUODENO-CHOLECYSTOSTOMY.*

BY J. MCF. GASTON, M. D., ATLANTA, GEORGIA.

In the numbers of this journal for September and October, 1884, I gave the details of my experiments upon six dogs, illustrating the fact that a fistulous communication between the gall-bladder and duodenum was practicable by means of a ligature through their walls. It was further shown that the inflammation in their tissues, from binding them together, induced a union of their surfaces around the site of the ligature, so that a fine septum existed in which there was a free opening from the gall-bladder into the duodenum.

With a view to verify those results, and to observe the effects of a further step, by ligation of the common bile duct, I have operated recently upon fourteen dogs, varying the proceeding as will appear in the report of these experiments—case No. 1 being undertaken in the forenoon of July 2, 1885. An old brindle dog was etherized, and an incision of five inches made obliquely to the right from the median line below the costal arch, exposing the gall-bladder and the duodenum, with the ducts.

A catgut ligature was tied upon the common bile duct, and a doubled white silk thread was passed, with a needle shaped like a fishhook, through such a portion of the walls of the gall-bladder and duodenum as to insure the entrance into their respective cavities, when it was tightened and knotted upon the suface of the duodenum.

The peritoneal membrane was closed with a continuous suture of fine catgut, and the external wound was united with interrupted suture of No. 11 Snowden's iron-dyed silk.

*Second series of cases.

Autopsy, July 5th : This old dog died last evening, having survived the operation only fifty-six hours. Upon the removal of stitches from the integument, no adhesion of the tissues existed; and upon removing the catgut suture within, the peritoneal lining was readily separated with the handle of the scalpel. There was considerable serous effusion in the abdominal cavity. The loop of silk thread still held the gall-bladder in juxtaposition with the duodenum, but without any agglutination of the adjoining surfaces. The ligation of the common duct remained, but from its cutting into the surrounding structures, included with the duct in the loop, was so loose, that an elastic tube directed from the duodenal opening passed through the canal within the ligature and went into the hepatic duct, thereby showing that cohesive inflammation had not united the walls of the common duct at the point of constriction. The elastic tube being carried from the gall-bladder through the cystic duct, passed into the common duct and out at the opening in the duodenum, thus verifying the fact that a flexible sound may traverse the cystic and common ducts. It was observed that under the distension of the gall-bladder without an outlet, its contents had made their way into the substance of the liver and escaped through its upper surface. The orifice of the common duct was found within an inch of the termination of the pylorus, and some two inches further on I discovered the opening of the pancreatic duct, being quite different from their close proximity usually observed in the human being, and even frequently entering the duodenum by one opening.

*Case No. 2*, in the afternoon of July 2, 1885.—This operation, done by Dr. W. D. Bizzell, at my request, on a young shaggy dark bitch. Being etherized, an incision was made below the ensiform cartilage, a little to the right of the median line, extending to the level of the umbilicus. The common duct was secured with a ligature of white silk, and a loop of the same was passed through the walls of the gall-bladder and duodenum, knotting it upon the surface of the latter. The peritoneum was closed with continuous suture of Snowden's iron-dyed silk No. 4, and the external wound with interrupted suture of iron-dyed silk No. 11, and compress with jacket completed the dressing.

Autopsy, July 4, 1885, by Dr. Bizzell. Care No. 2 having died during the night of the 3d, the examination showed that the gall-bladder was empty, and a collection of bile was found above the liver and in the thoracic cavity, indicating that it had permeated the hepatic tissues, from the disintegration of the structure of the adjacent layer of the sac. The common duct remained ligated and there were adhesions between the surfaces of the gall-bladder and duodenum around the site of the loop, but upon laying open the duodenum, it was found that the thread had not made an opening into the canal for the discharge of the bile, thus explaining the extravasation into the parenchymetous structure of the liver from the accumulation in the sac. There were little signs of any peritoneal inflammation, but the liver was thickened and softened from the burrowing of the bile.

*Case No. 3*, July 3, 1885.—In the forenoon a young yellow bitch, being etherized, I made an incision in the linea alba from the ensiform cartilage to the umbilicus, ligated common duct with catgut ligature, and passed a loop of white silk, doubled thread, through the walls of the gall-bladder and duodenum, with knot resting upon the latter. Closed peritoneum with continuous catgut suture and external wound with stitches of iron-dyed silk No. 11. Compress and jacket applied.

Autopsy, July 5.—Case No. 3 being found dead this morning, I found no union along the lines of suture in the abdominal wall. The gall-bladder was empty, and there was infiltration of bile into the substances of liver. The loop held the gall-bladder and duodenum together, but no opening was made by it for the discharge of the bile into the canal, so that it was forced through into the parenchymetous structure of the liver from the great accumulation in the sac, as noted in case No. 2.

The common duct was found occluded completely by the catgut ligature, so that a flexible sound was arrested at this point when directed from the orifice in the duodenum, or from the gall-bladder through the cystic duct. There were indications of considerable peritoneal inflammation in the abdominal cavity.

It should be stated that in this case, as in case No. 2, the loop of doubled white silk thread did not enter into the lumen of the duodenum, from failure to penetrate the cavity in passing the fish-hook needle ; and hence there was no division of the mucous membrane for the passage of the bile even partially into the canal.

*Case No. 4*, July 4, 1885.—An exceptionally good specimen of his species, in excellent condition, with cream-colored hair, being etherized, I made the oblique cut extending from the median line below the right costal arch about six inches. The common duct was ligated with catgut by the sense of touch, as it could not be seen, and the doubled silk thread was passed with the fish-hook needle through the walls of the gall-bladder and duodenum, making sure the entrance into their respective cavities.

Dr. —— Murray kindly closed the peritoneum with the continuous catgut suture and the external incision with interrupted suture of iron-dyed silk. The usual compress of old soft cotton cloth was placed over the wound with a little carbolized oil and a jacket sewed around the body of the dog.

Autopsy, July 8.—This animal, Case No. 4, died during the past night, having survived three and a half days, and upon opening the abdomen extensive inflammation of the abdominal viscera was present, with dirty serous effusion in the peritoneal cavity.

The stitch uniting the gall-bladder and duodenum had cut partially through the inclosed textures, but without any adherence of the adjacent surfaces. The common bile duct was effectually obstructed by the ligature of catgut, and the tissues were so much softened on either side of the constriction, that they yielded with very slight force in passing the flexible sound from the cystic and duodenal cavities. The disintegrating process had taken the place of adhesive inflammation, owing doubtless to the permeation of the liver by the bile.

*Case No. 5*, July 6, 1885.—To obviate the serious consequences of ligating the common duct while no outlet had been established for the bile, the following expedient was resorted to in a large, well-formed spotted bitch: Being etherized, the right oblique incision below the costal arch exposed the parts, and a loop of silk thread was passed deeper than in other cases through the walls of the gall-bladder and duodenum so as to approximate them and knotted loosely with the ends of the ligatures retained in the hand of an assistant to steady and fix the structures. The edges of each wall, projecting in a fold from this loop, lay in apposition, and a round piece was cut out of the gall-bladder and out of the duodenum by a shoemaker's punch, precisely opposite to each other. A continuous suture of fine catgut passed from the outside entirely around these circular margins attached them together, so that there existed an opening direct from the gall-bladder into the duodenum so as to let the bile flow out continuously.

Prior to punching out this hole in the gall-bladder, the contents were partially drawn off by aspiration, but a portion of thick bile remained that flowed out when the opening was made in its walls with the punch, which was in part taken up by sponges, and the rest was discharged at the external wound, and very little, if any, entered into the peritoneal cavity.

Dr. W. S. Elkin kindly assisted me in this operation and closed the peritoneum with catgut and the skin with iron-dyed silk thread.

Autopsy in Case No. 5, July 8.—This bitch died during the past night, surviving the operation about thirty-six hours. Neither the external nor internal suture had effected any agglutination of the parietes. Extreme peritonitis was present with softening of the hepatic structure, and the gall-bladder was nearly empty, but no satisfactory indications of the bile having passed into the duodenum. The continuous suture around the punched opening into the gall-bladder and duodenum had yielded at some points, admitting of the extravesation of bile into the peritoneum, and this without ligation of the common bile duct. Upon attempting to pass a problematic judgment in this case, it would seem that a mistake was committed in passing my securing loop so deeply into the tissues of the gall-bladder and the duodenum, and should the experiment be repeated hereafter, I shall only include the smallest portion of each that may suffice to hold them in contact while the holes are made and stitches of the interrupted suture, instead of the continuous order, are applied around their margins. The contractile tissues of the gall-bladder and duodenum tend to close the orifices made in their walls, which must loosen the continuous suture introduced under some distension, and hence, the interrupted stitches put in closely around the openings should secure more favorable juxtaposition of their margins.

*Case No. 6*, July 7, 1885.—A lean, tall, black dog being etherized I proceeded, in the presence of Drs. Divine, Wilson, Parks and Powell Walker, to repeat the steps taken in Case No. 5, with the addition of ligating the common duct with catgut. The peritoneal incision was closed with continuous catgut suture, and the skin with stitches of iron-dyed silk. A compress with a little carbolized oil was placed over the wound and a jacket sewed around the animal.

Autopsy of Case No. 6, July 8, 1885.—While there was no union in the skin, adhesion of the peritoneum had taken place, though the dog survived the operation only some eighteen or twenty hours. The circular continuous suture of catgut around the margin of the apertures, made by the punch in the walls of the gall-bladder and duodenum, had yielded, so as to admit of the free escape of bile into the peritoneal cavity, and although the retaining silk loop held the walls together there was no adhesion between these surfaces. The occlusion of the common duct was complete, as shown by passing the elastic sound from the gall-bladder and from the duodenum to the point of constriction by the catgut ligature. There was little evidence of peritonitis, notwithstanding the serous effusion mixed with bile in the cavity. The liver was brittle and apparently undergoing some disintegration, but was not materially enlarged.

*Case No. 7*, July 7, 1885.—A thick-set, yellow dog was etherized by Mr. Fite, and with the oblique incision below the right costal arch made by Dr. Powell Walker, I ligated the gall-bladder to the duodenum, tying the knot against the wall of the latter, so as to favor the escape of the loop into the canal. The closure of the peritoneum with continuous catgut suture and the external wound by stitches of iron-dyed silk was likewise done by Dr. Walker, after which the compress and jacket were applied.

July 12.—The jacket having slipped off the wound it was entirely removed, and it was soon found that the dog had torn all the stitches out, so that the external incision was gaping open, but revealed healthy granulations, and no further dressing was applied to Case No. 7.

July 24.—This animal has progressed in every respect well, and only awaits a favorable occasion for an explanatory opening with a view to verify the cholecysto-duodenal communication, and for the ligation of the common duct so as to throw all the bile into the gall-bladder, and thence through the newly-made fistulous opening into the canal of the duodenum.

*Case No. 8*, July 8.—A small, plump, black and tan dog being etherized, was operated upon by Dr. Powell Walker, at my request, and the steps corresponded with those adopted in previous case, excepting that fine white silk was used in the continuous suture for closing the peritoneal incision instead of catgut.

Autopsy of No. 8, July 9.—This dog lived some eighteen or twenty hours after the operation. There was no union of the integument, but the peritoneal incision had united. An abundant fluid exudation tinged with bile existed in the cavity, yet there was only slight indications of peritonitis. While the duodenum presented signs of inflammation, yet no adhesions had occurred between its surface and that of the gall-bladder. The bile had evidently escaped from the partial cutting of the wall of the sac by the ligature before any adhesive inflammation had been established for agglutinating it to the duodenum.

*Case No. 9*, July 8.—A shaggy-haired, yellow bitch being imperfectly etherized, was operated upon by Dr. Powell Walker, at my request, and from the struggling of the animal a large portion of the intestines protruded upon the table, but proceeded regularly in all other respects, as in Case No. 8, using the silk for closing the peritoneum.

July 24.—This animal eats well and is in excellent condition.

*Case No. 10*, July 9, 1885.—A black and tan bitch of medium size inhaled far more than the usual quantity of sulphuric ether, without being quieted, and there being great disturbance of the respiration that caused an apprehension of asphyxia, the ether was withdrawn entirely and the muzzle slackened on the mouth so as to allow a free entrance of atmospheric air, whereupon, the animal became composed, and the operation was performed by Dr. Powell Walker, at my request, apparently without any suffering of the subject. The right oblique abdominal incision, a single loop of doubled silk thread uniting the walls of the gall-bladder and duodenum, the closure of peritoneal incision by continuous suture of fine white silk, and union of integument by stitches of of iron-dyed silk, with compress over the wound and jacket around the body, constituted the measures adopted in this as in the preceding cases. This animal was found dead on the following day, but no *post mortem* examination was made.

*Case No. 11*, July 11, 1885.—A thick-set, spotted old dog being etherized, was operated upon by Dr. Powell Walker, at my request, as detailed in Case No. 10 and previous cases, simply verifying the experiments performed last year, hoping that some of the subjects may survive, so as to ligate the common duct subsequently.

Autopsy in Case No. 11, on July 13, 1885.—The external wound was not united by adhesive inflammation, though the stitches remained intact, while the peritoneal union was completed within the forty-eight hours that this animal survived. There were extensive adhesions around the site of the ligature in the gall-bladder and duodenum, and it was found requisite to detach the right lobe of the liver from the latter. The surface of the gall-bladder being adherent to the surface of the duodenum, which being broken up showed an opening by the cutting of the ligature through the adjacent tissues, so that a communication would ere long have been established between the cavities of the gall-bladder and duodenum, with a soldering of their surfaces around the ligature.

It was observed that such a portion of the wall of the sac had been included in the loop as to cause some folding on itself, and it was crumpled up by the close constriction of the ligature. There was no indication of escape of bile into the cavity of the peritoneum, nor were these signs of general peritonitis or of any material alteration in the texture of the liver.

Instead of disintegration, as observed in other cases, the process of adhesive inflammation was set up in the tissues, so that a favorable issue might have been anticipated if the acute inflammation had subsided.

*Case No. 12*, July 12, 1885.—A young, yellow dog, of medium size, behaved, under the inhalations of sulphuric ether, much after the manner of Case No 10, and as casually referred to in Case No. 9, and the animal remained quiet after its withdrawal.

At my request, Dr. Powell Walker operated as in the foregoing cases, with the exception that the interrupted suture of white silk was passed jointly through the peritoneal and cutaneous incisions of the parietes.

July 14.—The jacket around the body of this animal was torn off during the night, but the wound is not gaping, and hence it is not replaced.

July 24.—Case No. 12 is progressing favorably, and eats raw beef or other solid food eagerly, giving an assurance of affording an opportunity to ligate the common duct in a few days.

*Case No. 13*, July 13, 1885.—A yellow bitch being only partially etherized, at my request Dr. Wile made an incision in the linea alba from the ensiform cartilage to the umbilicus, when Dr. F. H. Peck passed the doubled white silk ligature through the walls of the gall-bladder and duodenum, with the knot resting on the latter. The peritoneal incision was closed with continuous suture of fine iron-dyed silk, No. 3, and cutaneous incision with interrupted suture of coarse iron-dyed silk, No. 13, compress and jacket.

*Case No. 14*, July 13.—A lean, shaggy, yellow dog was partly etherized, and Dr. Peck, at my request, made an incision in the linea alba, extending from the xiphoid cartilage below the umbilicus, and secured the walls of the gall-bladder and duodenum together by a loop of doubled white silk thread, knotting it upon the surface of the latter. The lining membrane was closed with a continuous suture of fine iron-dyed silk, and the integument with interrupted suture of coarse iron-dyed silk. A compress was applied over the wound and a jacket sewed around the body of the animal.

It was noted in this case, as in several others, that there was less muscular movements of the animal after withdrawing the sulphuric ether than during its inhalation, and there seemed to be no consciousness of suffering in this state.

Autopsy in case No. 14, on July 14.—The dog lived but twenty-four hours, and the cause of death was doubtless in part the element of shock. There were signs of some inflammation in the duodenum, and serous effusion in the cavity of the abdomen, but no adhesion between the surfaces of the gall-bladder and duodenum around the site of the ligature, which was verified to be at the proper distance from the pyloric orifice of the stomach. The location of the orifices of the common duct and the pancreatic duct were nearly two inches apart in the duodenum.

It will be noted that there are four dogs living, July 24th, 1885, and that these have survived the operation of simply ligating the gall-bladder to the duodenum, respectively, 17, 16, 12 and 11 days, with good prospects of complete recovery from this proceeding, and with almost the certainty of having a fistulous opening between the cavities of the gall-bladder and duodenum at the present time. Within one month or less from the date of each experiment, it is my intention to reopen these animals, and ligate the common bile duct, so as to turn the bile entirely through the artificial communication from the gall-bladder into the duodenum, thus effecting what I had hoped to do with the subject of my sixth experiment last year, that died from the etherization on the occasion of a third laparotomy for this purpose.

The results of these final experiments must be reserved for the next number of THE JOURNAL.

